# The Cognitive Neuroscience of Stable and Flexible Semantic Typicality

**DOI:** 10.3389/fpsyg.2019.01265

**Published:** 2019-05-31

**Authors:** Jonathan R. Folstein, Michael A. Dieciuc

**Affiliations:** Department of Psychology, Florida State University, Tallahassee, FL, United States

**Keywords:** typicality, categorization, semantic cognition, situated cognition, cognitive neuroscience

## Abstract

Typicality effects are among the most well-studied phenomena in the study of concepts. The classical notion of typicality is that typical concepts share many features with category co-members and few features with members of contrast categories. However, this notion was challenged by evidence that typicality is highly context dependent and not always dependent on central tendency. Dieciuc and Folstein (2019) argued that there is strong evidence for both views and that the two types of typicality effects might depend on different mechanisms. A recent theoretical framework, the controlled semantic cognition framework (Lamdon Ralph et al., 2017) strongly emphasizes the classical view, but includes mechanisms that could potentially account for both kinds of typicality. In contrast, the situated cognition framework (Barsalou, 2009b) articulates the context-dependent view. Here, we review evidence from cognitive neuroscience supporting the two frameworks. We also briefly evaluate the ability of computational models associated with the CSC to account for phenomena supporting SitCog (Rogers and McClelland, 2004). Many predictions of both frameworks are borne out by recent cognitive neuroscience evidence. While the CSC framework can at least potentially account for many of the typicality phenomena reviewed, challenges remain, especially with regard to *ad hoc* categories.

## Introduction

The idea that category membership is a matter of degree was proposed by the philosopher Wittgenstein (1953), and became prominent in cognitive psychology with the seminal studies of Rosch and colleagues (Rosch and Mervis, 1975), who referred to graded membership as “typicality.” Observers rate some concepts as better members of their category than others, place typical exemplars in their categories more quickly and easily than atypical exemplars, and assign typical exemplars to a category more consistently than atypical exemplars, which are more likely to be assigned to contrast categories (Rosch and Mervis, 1975). All of these findings can be interpreted in terms of ease of conceptual access: typical exemplars are retrieved and recognized more easily than atypical exemplars. Typicality is now accepted as a ubiquitous phenomenon that must be accounted for by any adequate theory of category membership or semantic cognition.

The now classic interpretation of typicality effects is rooted in similarity, which can be expressed in terms of number of features shared between two representations or in terms of their distance from each other in a continuous psychological space (Shepard, 1964; Tversky, 1977). Across a number of studies, Rosch provided evidence that typical exemplars of categories were more similar to the members of their category and less similar to contrasting category members than atypical exemplars (Rosch and Mervis, 1975). Rosch’s similarity account was (and still is) consistent with several theoretical models of classification that leverage similarity, including exemplar-based models (Nosofsky, 1984; Kruschke, 1992), prototype-based models (Smith and Minda, 1998), hybrids of the two (Love et al., 2004), general recognition theory (Ashby and Maddox, 1993), and connectionist models (Rumelhart and Todd, 1993). All of these classes of models remain relevant today and continue to be useful in predicting categorization phenomena.

Importantly, however, challenges to the similarity-based account of conceptual structure arose in the 1980s that emphasized the dependence of typicality on contextual constraint (e.g., Barsalou, 1983; Roth and Shoben, 1983). One very broad characteristic of these challenges was that typicality is very flexible or “unstable” in the sense that it can be changed within an individual *via* manipulation of various types of context. The critical mechanism proposed to cause this instability is that typicality effects are not caused by similarity of an exemplar to other members of the same or different category, but by “fit” between an exemplar and a particular context. Consistent with this idea of fit, Murphy and Medin (1985) argued that top-down knowledge structures – such as schemas, theories, and knowledge – were important determinants of category membership. Thus, category members that are atypical by similarity-based metrics might fit a particular situation far better than more typical exemplars. For instance, a penguin, an atypical bird for most people, fits a scene from Antarctica, or even a zoo, better than a generally more typical robin.

In a recent review (Dieciuc and Folstein, 2019), we argued that convincing evidence exists for both similarity-based and contextually based typicality, proposing the terms “structural typicality” for the classical Roschian type and “functional typicality” for the Barsalounian type (Barsalou, 1987; Yeh and Barsalou, 2006). We also proposed that the two kinds of typicality effects are driven by different mechanisms. Structural typicality effects are caused by access to a long-term conceptual store containing representations organized by classical Roschian similarity. Functional typicality effects are caused by processing of semantic information in working memory, which will often require integration of target concepts into situational contexts. Finally, we noted two theories that are broadly consistent with this framework, but differently emphasize the two types of typicality: situated cognition (henceforth, “SitCog”; Barsalou, 2003, 2009a,b), which emphasizes the importance of unstable functional typicality effects, and the controlled semantic cognition framework (henceforth, “CSC”; Lamdon Ralph et al., 2017), which emphasizes stable structural typicality effects.

In the current paper, we evaluate these two large theoretical frameworks in their ability to account for structural and functional typicality effects, focusing primarily on data from cognitive neuroscience. The paper is organized as follows. First, we briefly review the key properties of SitCog and CSC that result in predictions for neural representations supporting structural and functional typicality. We then evaluate the predictions made by the two theories. Finally, we evaluate the ability of the computational models associated with CSC, which have been extensively developed by Rogers, McClelland, and colleagues, to predict the typicality effects reviewed in our previous paper. We focus here on phenomena related to functional typicality because the model is obviously well suited to account for structural typicality effects. Whereas few functional typicality effects have been explicitly simulated by the model, our emphasis here is on the ability of the model’s architecture to represent the kinds of contextual constraints we hypothesized to drive functional typicality effects. We conclude by suggesting challenges to both the SitCog and CSC frameworks moving forward.

## The Controlled Semantic Cognition Framework

The *controlled semantic cognition* (CSC) framework is a broad theory proposing a network of brain areas serving access to and use of semantic information. The theory and associated models have been used to account for a wide range of evidence, including cases of category-specific deficits from brain damage (Rogers et al., 2004; Chen et al., 2017), the order in which categories are acquired in childhood and lost due to brain damage (Rogers and McClelland, 2004; Rogers and Patterson, 2007), and the loss of semantic information across multiple modalities resulting from semantic dementia (Lambon Ralph et al., 2010; Mayberry et al., 2011). The CSC framework proposes two major components to the network: (1) a network of brain regions that stores conceptual information and (2) a network of brain regions that controls how a subset of that conceptual information gets recruited in a context- and task-dependent manner.

**Figure 1:**
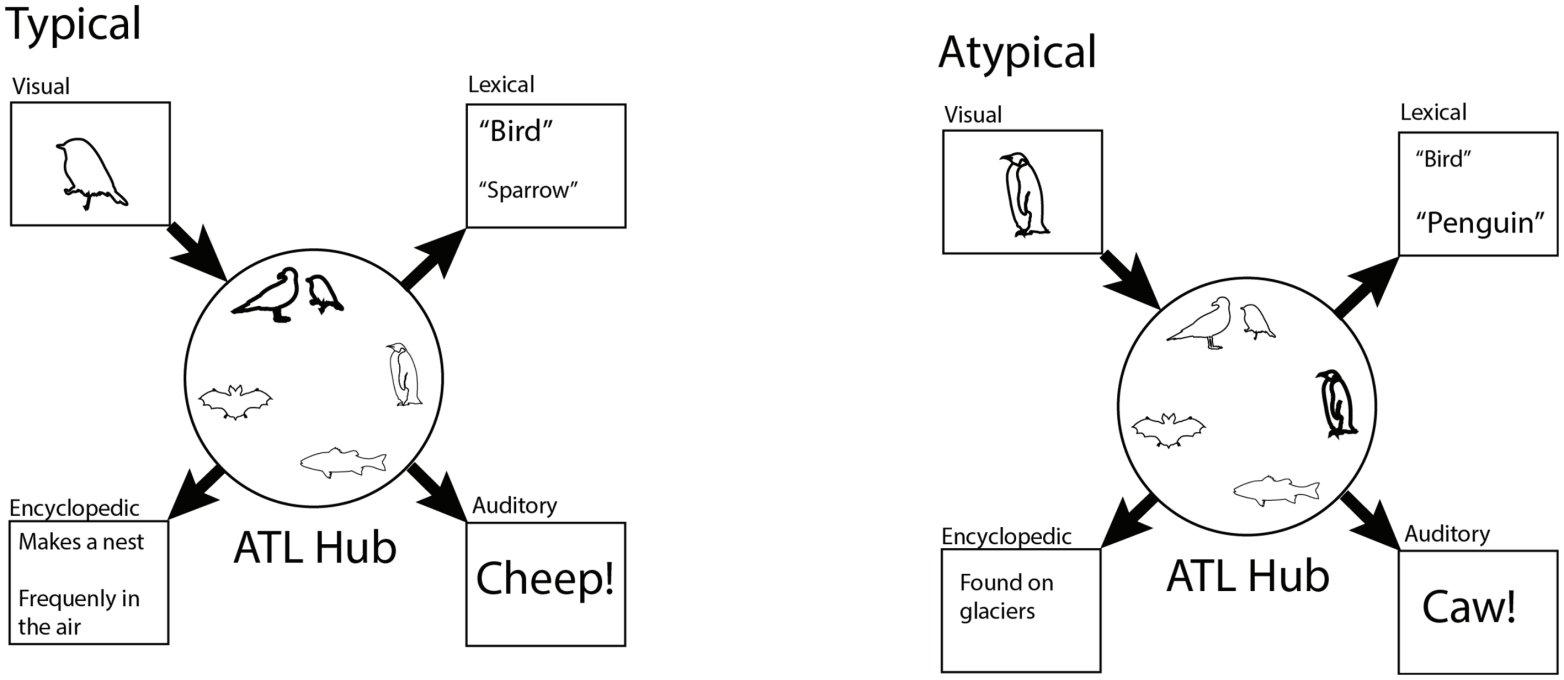
Cartoon illustration of the hub and spoke model. The hub (central circle) represents stable similarity relations between concepts based on the semantic features that each concept activates. In the left-hand panel, a sparrow is used as input. A sparrow is a typical bird, because it has similar features to other birds. The representation for the name “bird” is amplified because it is reinforced by other similar birds. In the right-hand panel, the less typical penguin is used as input. The “bird” label is more weakly activated because other birds are dissimilar to penguins.

The first component, described by the “hub and spoke model,” illustrated in Figure 1, is a network of brain regions that is responsible for storing conceptual information. The spokes of this network are composed of modal regions of the brain that predominantly process one type of sensory, motor, affective, or interoceptive information. In contrast to the spokes, the hub is located in the anterior temporal lobe (ATL), responds to information regardless of what modality it was presented in, and is hypothesized to facilitate associative links between the modality-specific areas. For instance, a picture of a dog is passed from the visual spoke, through the hub, which allows us to access semantic features about how the dog feels and sounds. Lamdon Ralph and colleagues have argued that the hub constitutes a kind of conceptual core that contains information distilled across multiple episodes: “[The hub ensures that] the same core information is activated each time an entity is encountered even if different aspects occur in separate episodes.” (Lambon Ralph et al., 2010).

Several specific computational models have been used to simulate the effects of damage to the hub (Rogers et al., 2004; Rogers and McClelland, 2004; Rogers and Patterson, 2007; Chen et al., 2017). The hub is modeled as an intermediate “representation” layer in a PDP model that mediates associations between input and output layers sometimes meant to correspond to modality-specific cortical areas (i.e., the “spokes”). The representation layer develops distributed representations for each concept that emerge as a result of learned associations between representations in the modality-specific spokes (e.g., Chen et al., 2017) or between input and output representations (Rogers and McClelland, 2004). The similarity between conceptual representations in the hub layer is determined by the overall similarity between their modality-specific representations – for example, similarity between dog and sheep representations is determined by similarity between visual, auditory, motor, somatosensory, and linguistic features of dogs and sheep, considered as a whole. Importantly, the hub, or representation layer, is not modulated by context and thus represents invariant patterns with similarity determined by the shared features of concepts. As we will see, these properties allow the model to easily predict classical Roschian typicality effects.

The second component of the CSC framework, called the control network, is hypothesized to correspond to a network of prefrontal and temporoparietal regions, damaged in a disorder called semantic aphasia. The network serves functions related to cognitive control, including modulating which information is recruited from the hub and spokes for a given task, selecting among concepts that are very similar, and suppressing strongly associated but contextually inappropriate information (Jefferies, 2013).

**Figure 2:**
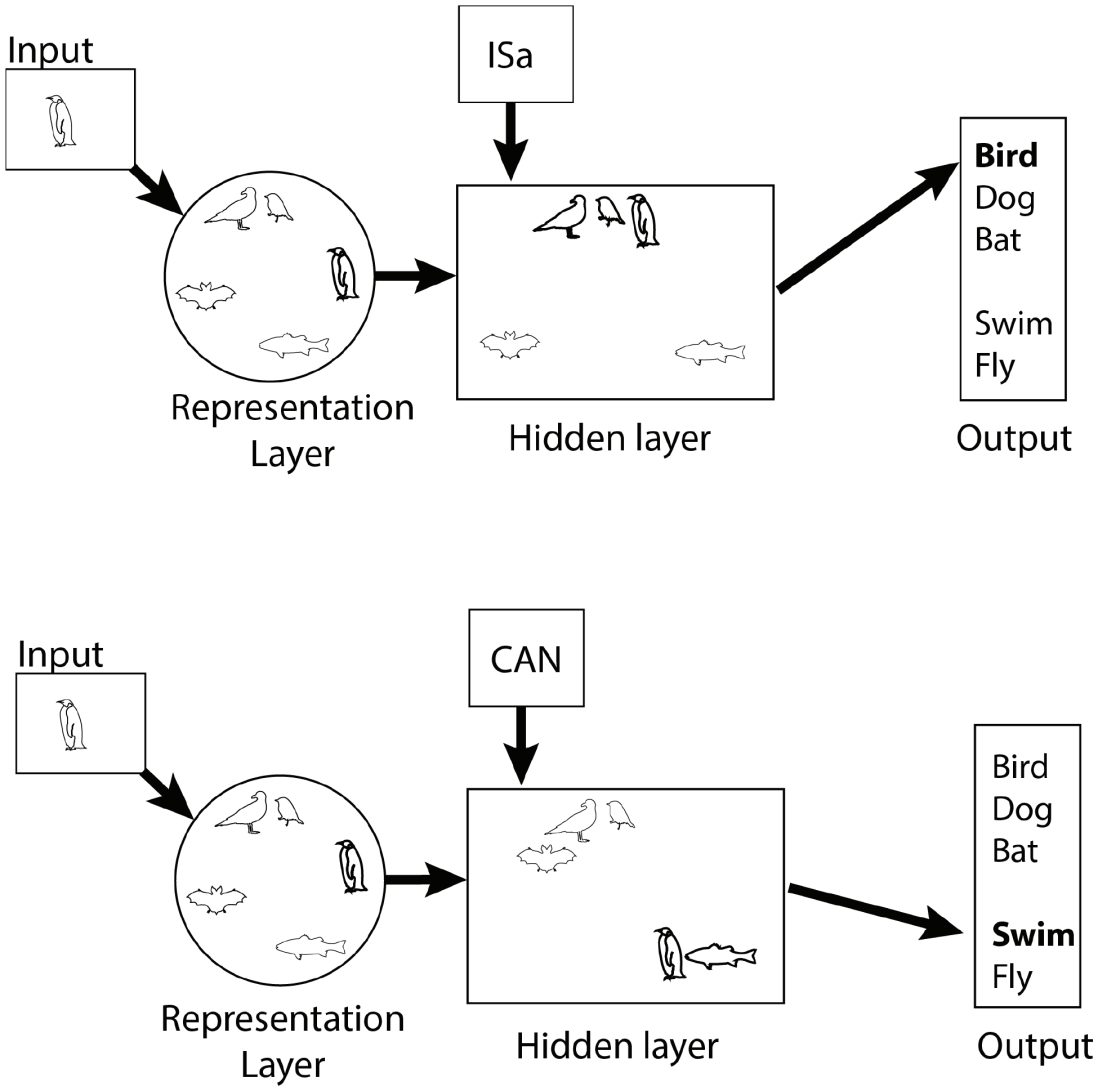
Cartoon illustration of the Rumelhart model. The representation layer has the same properties as the hub, shown in Figure 1. Similarity within the hidden layer changes depending on the desired semantic information. Penguins are similar to other birds if naming is required (top, ISa context) but more similar to fish when functional properties are required.

The control network has been less extensively explored in computational models than the hub, but it is linked to the Rumelhart model (Rumelhart and Todd, 1993; Rogers and McClelland, 2004). This model, illustrated in Figure 2, has four layers: an input layer with input nodes corresponding to objects, like sparrow, dog, etc.; an output layer with semantic features associated with the input; and two intermediate layers – the representation layer and the hidden layer. In the simplest applications of the model, activation is fed forward from the input layer, to the representation layer, to the hidden layer, and then to the output layer. The representation layer is the computational analog to the hub, described above, while the hidden layer is the analog of the control network.

The hidden layer is modulated by relational context nodes, which select what semantic output is required by the task. The context nodes are connected only to the hidden layer with weights trained by back propagation. They are distinct from the representation layer, which also sends activation to the hidden layer, because they are activated by the homunculus, who knows what kind of semantic information is needed, rather than by weighted connections from the input layer. The four nodes correspond to IS (produces adjectives like “tall” or “alive”), ISa (produces labels, like “bird” or “cat”), HAS (produces features like “wings” or “skin”), and CAN (produces actions, like “fly” and “bark”). For instance, if “cat” is activated in the input layer and HAS is activated in the context layer, the output layer might produce “claws,” “whiskers,” “fur” and whatever other features the network is trained to associate with cats. Critically, similarity between representations in the hidden layer is determined by the context layer. If the CAN node is activated, representations in the hidden layer of things that “can” do the same things will be very similar. For instance, things that can fly like bats and birds will tend to be more similar to each other than they are to things that cannot fly, like dogs and penguins. Thus, whereas the representation layer represents similarity the same way in every context, similarity within the hidden layer is context dependent.

An important property of the Rumelhart model and other related models is that they are very well suited for predicting structural typicality effects because their representations are fundamentally sensitive to similarity based on shared semantic features. Because the model’s representations change with context, it also offers a potential account of functional typicality.

Importantly however, the effect of context on the model is, at least *prima facie*, driven by alteration of similarity between object representations. Thus, context changes typicality of a given representation in the hidden layer because it changes the distance of representations from the central tendency of the nearest cluster (Rogers and McClelland, 2004, Chapter 7, Figure 7.2). Also, context alters typicality through specification of the kind of information that is requested, presumably because it is important for a given task. Thus, typicality in the hidden layer changes depending on whether one wants to know a name (ISa), what actions the object can perform (CAN), or what the object looks like (IS).

As will prove important in later sections, there is more than one way that “knowledge” represented by the Rumelhart model can be assessed. We mentioned above that activation can be fed forward from the input layer through the representation and hidden layers, and out to the output layer, which represents semantic features. However, the model is also capable of receiving activation starting in the output layer. In this procedure, called “back propagation to activation,” one or more semantic features are activated in the output layer and a distributed pattern of activation is found in the representation layer that maximally activates the chosen semantic features. For instance, one can find the representation layer pattern that activates “HAS fur.” Once this pattern is found, the model can be queried about what other features it activates. Thus, it might be found that the same pattern that activates “HAS fur,” also activates “CAN walk,” “HAS skin,” and “HAS legs,” as most things with fur do (Rogers and McClelland, 2004, Chapter 6). This ability of the model to query associations between features will be important when considering effects of context on typicality that seem to challenge the CSC framework.

Taken together, the neural and computational aspects of the CSC make two major predictions that we wish to evaluate in this paper. Both predictions stem from the larger prediction that concepts should be represented in at least two separate “neural theaters,” one sensitive to context and the other insensitive to context^1^. This prediction is generated by the observation that semantic dementia and semantic aphasia affect different anatomical areas and result in qualitatively different conceptual deficits and by the architecture of the Rumelhart model, which has separate context-sensitive and context-insensitive layers.

First, the context-insensitive neural theater, presumed to be located in the ATL, should contain representations of concepts for which similarity to the central tendency of their category should be correlated with standard ratings of typicality. A closely related prediction is that similarities between conceptual representations in this neural theater should mirror similarities between concepts as determined by standard feature listing tasks (e.g., De Deyne et al., 2008). Much evidence supporting the first two predictions has already been reviewed in support of the CSC framework (Lamdon Ralph et al., 2017). We briefly revisit some of this evidence and also add further evidence from fMRI.

The second prediction is less strongly emphasized in reviews of the CSC framework and is inspired by the hidden layer in the Rumelhart model. It is that the context-sensitive neural theater should still represent individual concepts, but similarity between the concepts should change depending on the type of information required by the task. This prediction is consistent with the idea that “functional typicality” as described by Dieciuc and Folstein (2019) can change depending on context, but only applies to cases in which those effects are driven by differential weighting of some types of information more than others due to contextual constraints. For instance, a piano’s weight is emphasized in a moving/lifting context whereas its sound is emphasized in a musical context (Barclay et al., 1974). In the former case, piano is an atypical instrument because it is dissimilar to other instruments in weight but a more typical piece of furniture because many pieces of furniture are very heavy. In the latter case, where sound is emphasized, the piano is a more typical instrument and not similar to furniture at all. An implementation of the Rumelhart model that represented these two contexts (e.g., “feels like” vs. “sounds like”) would plausibly represent pianos and couches as similar in the hidden layer in the moving/lifting context and the pianos and xylophones as more similar on the musical context.

Below we argue that different mechanisms, less clearly available in the Rumelhart model, are necessary to accommodate a separate set of cases in which contexts serve as better cues for some concepts than others, such as the finding that milk is more typical than tea for a doughnut shop but the reverse is true for an afternoon break (Roth and Shoben, 1983).

### Evidence for Neural Theaters of Context-Invariant Representation

The classical account of typicality proposed by Rosch and colleagues, which we call structural typicality, posits that typical objects share more attributes with members of their category and fewer attributes with nonmembers than atypical members (Rosch and Mervis, 1975). The ability to account for Roschian typicality effects is fundamental to the design of the hub and spoke portion of the CSC framework because the internal representations formed in the hub are predicted by computational models to cluster by similarity: the more modality-specific features in the “spokes” shared by two concepts, the more similar their internal representations (Rogers and McClelland, 2004; Rogers and Patterson, 2007). Basic level categories tend to form tight clusters in which objects are more similar to each other than to other basic level categories and typical exemplars occupy central regions of the clusters because they share features with many members of the category (Rosch et al., 1976; Rogers and Patterson, 2007). These properties allow the Rumelhart model to predict a classic behavioral finding related to typicality. Briefly, the finding relates to the effect of typicality on naming at the basic level (bird vs. fish) compared to subordinate level (sparrow vs. penguin). Observers name typical items more quickly at the basic level and atypical items more quickly at the subordinate level; thus, sparrows are called birds most quickly and penguins are called penguins most quickly (Rogers and McClelland, 2004, Chapter 5). The model predicts this because sparrows have many similar neighbors that all activate the “bird” output label when the sparrow is input to the model. Penguins have fewer similar neighbors and activate the bird label less strongly. For similar reasons, the model correctly predicts that basic level names are learned faster for typical than atypical items (Rogers and McClelland, 2004, Chapter 5).

The computational properties of the hub predict a neural theater with context-invariant representations and stable similarity relations supporting structural typicality and resulting effects. We now review evidence from neuropsychology and neuroimaging supporting this prediction.

#### The Anterior Temporal Lobe

Evidence from semantic dementia, already reviewed elsewhere (Lamdon Ralph et al., 2017), suggests that the anterior temporal lobe hosts just such a neural theater. In naming tasks, exemplars rated as typical are more resistant to damage than less typical members because their internal representations are similar to many other concepts from the same category. This often results in correct naming even when, as a result of damage, a somewhat incorrect internal representation is activated (Lambon Ralph et al., 2010). This is predicted by the Rumelhart model because damage to internal representations is predicted to cause “representational distortion” that in turn causes concepts to be confused with different similar concepts (e.g., Rogers et al., 2015). Patients with SD categorize typical exemplars more accurately than atypical exemplars and, in drawing tasks, tend to omit atypical or distinctive features and include shared, typical features. SD patients also make informative confusion errors: exemplars are incorrectly assigned to categories with which they share typical features, for instance categorizing butterflies as birds (Bozeat et al., 2003; Mayberry et al., 2011; Rogers et al., 2015). Data from Transcranial Magnetic Stimulation (TMS), in which the ATL is temporarily suppressed, further corroborate these findings. TMS applied to the ATL increases the time required to name atypical but not typical exemplars (Woollams, 2012).

Impressive recent successes enjoyed by the Roschian approach to conceptual structure have also come in neuroimaging studies of healthy participants. In these studies, typicality, assessed in the usual acontextual manner, is shown to be predictive of the neural structure of knowledge. Iordan et al. (2016) showed that typicality ratings predict central tendency in neural representations of objects in brain areas that subserve high-level vision. fMRI voxel patterns elicited by exemplars of several object categories were averaged to create prototype patterns for each category. The similarity between the fMRI pattern for each exemplar could then be compared to the prototype pattern for its category. Exemplars rated as more typical elicited voxel patterns that were more similar to the prototype than exemplars rated as less typical. It is reasonable to expect that perceptual features make a large contribution to the neural representations in the visual brain areas observed in this study (e.g., Freedman et al., 2003; Jiang et al., 2007, but see below), consistent with the Roschian view of typicality as driven by stable features of objects (Rosch and Mervis, 1975).

A virtual explosion of recent papers have reached similar conclusions relating abstract semantic features to neural representations in more multimodal cortical areas not directly related to perception. These studies did not collect typicality ratings directly but, consistent with the Roschian view of feature sharing, used feature norms from studies in which participants generated semantic features in response to concept cues such as “bird” or “dog.” Semantic similarity between concepts was characterized based on the number of features shared between concepts and these similarities were in turn compared to similarities between fMRI voxel patterns elicited by the concepts when processed by the participants in the scanner, a technique called “representational similarity analysis,” or RSA (Kriegeskorte et al., 2008). The anterior temporal lobe, specifically including the perirhinal cortex and the more anterior temporal pole, emerged as a particularly important area where semantic similarity between concepts matched similarity between voxel patterns elicited by conceptually processed pictures (Bruffaerts et al., 2013; Fairhall and Caramazza, 2013; Clarke and Tyler, 2014; Borghesani et al., 2016; Chen et al., 2016) and words (Bruffaerts et al., 2013; Fairhall and Caramazza, 2013; Liuzzi et al., 2015; Borghesani et al., 2016). Whereas these studies did not specifically address typicality, they support the claim that categories have stable central tendencies as the Roschian view suggests, and that these central tendencies are represented in the ATL.

The most parsimonious interpretation of studies linking similarity between neural representations with similarity based on participant ratings is that they demonstrate stable representations of similarity. However, in our view, it is important not to be overly sanguine in assuming total insensitivity to context. It has been argued, for instance, that typicality ratings are less consistent than implied by Rosch’s original studies, which reported between subject correlations as high as 0.91 (Rosch and Mervis, 1975; Barsalou, 1983, 1987; Barsalou and Sewell, 1984). Barsalou and colleagues argued that these analyses were distorted because the tests used were sensitive to the central limit theorem such that large numbers of participants inflated the size of the correlations. More appropriate analyses showed that typicality judgments in isolation were much less consistent (0.45 between people) and that consistency rose considerably when context was specified (Yeh and Barsalou, 2006). Yeh and Barsalou (2006) speculated that the inconsistency was observed because participants often judged conceptual typicality relative to situations that they called to mind, which were not always consistent.

The implication of these results is that it is difficult to know the effect of context on a dependent measure unless one manipulates context. Whereas several studies reviewed above have demonstrated representation of typicality or semantic similarity in the ATL, none have manipulated semantic context^2^, leaving open the possibility that manipulation of context could account for additional variance in representational similarity. As a case in point, consider the study reviewed above by Iordan et al. (2016), which demonstrated that typicality ratings accurately predicted similarity between a neural representation of an object and the average neural representation of the object’s category. Even though this study suggested that object representations in the lateral occipital complex (a visual area) supported Roschian typicality, other studies, reviewed below, have shown at least some effects of semantic task context in this or similar visual areas (Harel et al., 2014; Bracci et al., 2017).

Thus, correlations with feature ratings or typicality ratings taken in the absence of context do not necessarily force an interpretation of context insensitivity.

### Evidence for Context-Sensitive Neural Theaters Consistent with the Controlled Semantic Cognition framework

The studies reviewed in the previous section support the prediction that the ATL supports a context-insensitive neural theater representing stable similarity relationships between concepts. Importantly, however, the usefulness of a concept’s semantic features differs across situations – e.g., the front part of a hammer is useful when hammering in a nail, the claw part of a hammer is useful when trying to remove a nail from a board, and the length of a hammer is useful when trying to fish a toy from under a couch. A wide range of evidence, reviewed by Yeh and Barsalou (2006), suggests that semantic features are selectively represented when they are useful, giving them greater weight in decision-making.

In the Rumelhart model, access to context-appropriate features is mediated by a hidden layer in which similarity between representations changes depending on the type of information required by the context (ISa, IS, HAS, and CAN). This hidden layer generates the prediction of a neural theater in which similarity between representations changes depending on the demands of a given task.

#### Visual Cortex

One potential neural theater for this type of context is visual cortex, where task-dependent changes in similarity have been observed at multiple timescales. A seminal fMRI study by Li et al. (2007) had participants categorize a set of artificial stimuli that differed along continuous dimensions of shape and motion. When participants categorized the stimuli according to how they moved, distributed neural patterns in visual cortex elicited by stimuli that differed in motion were less similar than patterns elicited by stimuli that differed in shape. When participants categorized according to shape, the pattern was reversed: stimuli that differed in shape had less similar patterns than stimuli that differed in motion (see also van der Linden et al., 2013). Using fMRI adaptation as a measure of neural similarity, Folstein et al. (2013) showed that these effects of category learning were apparent even when participants were not actively categorizing the stimuli. Morphed cars that differed along a category-relevant dimension were less neurally similar than morphed cars that differed along a category-irrelevant dimension when participants performed a location detection, rather than a categorization task (see also Dieciuc et al., 2017).

#### Frontal and Parietal Cortex

The CSC framework emphasizes the role of frontal and parietal cortices in selecting contextually appropriate information based on evidence from a disorder called semantic aphasia (SA), a disorder caused by atrophy to frontal and temporoparietal areas, which are relatively spared in in SD. Findings from SA suggest that these areas are important for inhibiting prepotent semantic information in favor of task-appropriate information. Patients with SA are easily misled by strong semantic associates, for instance indicating that a synonym for “Piece” is “Cake” (a strong semantic associate) rather than “Slice” (Noonan et al., 2010) and choosing “mouse trap” (an associate of “fly swatter”) rather than “newspaper” as something to use to kill a fly (Corbett et al., 2011). The latter study also suggests that the ability of SA patients to use context for selection is weakened, such that stronger contextual cues are required to retrieve the correct information. Performance of SA patients asked to mime actions associated with objects was facilitated by context cues, including pictures of the object and holding the actual object, while controls were at ceiling in all conditions.

Whereas these studies suggest that frontoparietal cortices are important for retrieving appropriate information, they do not directly support the prediction of a neural theater in which context alters similarity between semantic representations, in turn altering which representations are most typical. Several recent fMRI studies, in which representational similarity analysis was used to measure neural similarity between objects while participants processed them in contrasting semantic tasks, confirm this prediction. In one study, which we highlight as particularly relevant to our concerns, participants judged in a 1-back task whether handheld objects belonged to similar semantic categories or were manipulated by similar hand motions (Bracci et al., 2017). In prefrontal, parietal, and high-level visual cortices, neural similarity between objects was better predicted by shared category than shared action during the semantic judgment task, but better predicted by shared action than shared category in the action judgment task. The pattern was much stronger in prefrontal and parietal areas than visual areas, however, which were most sensitive to perceived shape, somewhat sensitive to action during action judgments, and insensitive to semantic category. In contrast, prefrontal and parietal areas contained almost no information about task-irrelevant features. Other recent studies have come to very similar conclusions using very similar methods: neural similarity between objects represented in prefrontal and parietal cortices is almost completely dependent on task (Harel et al., 2014; Erez and Duncan, 2015; Bugatus et al., 2017).

Under the assumption that distance from central tendency determines or strongly influences typicality, it can be inferred that typicality of objects as represented in these areas is dependent on context as well.

## The Situated Cognition Framework

The SitCog framework has much in common with CSC. Like CSC, SitCog posits that semantic cognition is the result of an interaction between long-term memory and working memory (i.e., control structures). Also like CSC, SitCog posits that semantic knowledge is represented by reactivating perceptual cortex (the “spokes”) *via* long-term memory representations (the “hub”).

**Figure 3:**
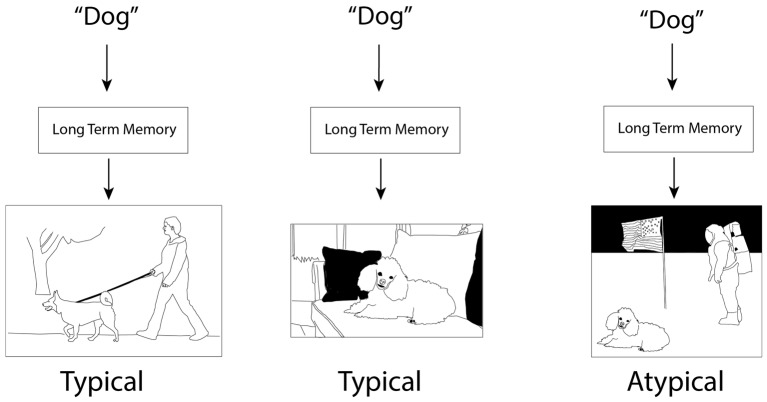
Cartoon illustration of the situated cognition framework. Input, including task requirements and, in this case, words, results in situation simulations, not only of the concept, but of a context in which the concept is embedded. Typicality judgments are driven by the match between concept and context. Note that the simulation poodle in the third panel is possible (cf. Barsalou, 1999) and was indeed required for the creation of the figure, but it is unlikely when the word “dog” is a cue, rendering dogs far less typical in this context than the other two contexts. Note also that the posture of the dogs in the first two panels (standing vs. sitting) is determined by the context rather than the frequency or “averageness” of the respective postures, reflecting sensitivity to causal interactions within situations.

The theories differ in how conceptual knowledge is represented. In contrast to CSC, which treats *features* as the most important component of concepts, SitCog emphasizes *situations* as the most important component of concepts (Barsalou, 2009b; see also Yee and Thompson-Schill, 2016). Situations are construed somewhat broadly and can vary in their complexity, but are often episodes of the type that are encoded into episodic memory. These include visuospatial scenes and events (Figure 3) as well as their accompanying internal states such as emotion and interoception. The central purpose of semantic long-term memory is to predict the contents of situations by producing “situated simulations,” or perceptual reenactments of situations for various contextually appropriate purposes (Barsalou, 2003).

Situations are fundamental to representations of individual concepts. Knowledge of a concept like “tent” constitutes the ability to simulate situations that include tents, such as circuses, campsites, and possibly outdoor weddings. When retrieving knowledge of dogs, one does not simply retrieve barks, fur, and tails, but simulations of dogs walking in parks, hunting in the woods, and, for some people, sitting on couches in New York apartments. Importantly, each of these situations will contain a different dog appropriate for the situation (and likewise for the tent examples above), for example, a golden retriever for the park, a bloodhound for the woods, and a toy poodle for the apartment.

Our major concern is SitCog’s account of typicality effects, including general ease of conceptual access and typicality ratings. Whereas ease of access to concepts in traditional frameworks, including CSC, is determined by similarity to fellow category members, ease of access in SitCog is determined by ease of integration into a situation. Thus, chickens might be typical birds for a farm whereas penguins are typical birds for a glacier and each will be more easily recognized and processed in their appropriate context (Yeh and Barsalou, 2006). In our recent paper on functional and structural typicality (Dieciuc and Folstein, 2019), we review several lines of evidence cited in support of SitCog, including ratings of typicality in *ad hoc* categories (Barsalou, 1983, 1985), changes in typicality ratings depending on the perspective taken by the observer (Barsalou and Sewell, 1984), changes in typicality ratings depending on the context of the rated object (Roth and Shoben, 1983; Freeman, 2014), and use of ideals rather than central tendency for typicality ratings, ideals being a measure of usefulness for a task or goal (Barsalou, 1985; Lynch et al., 2000; Burnett et al., 2005).

In the current paper, we focus on the predictions of SitCog for neural data specifically relating to functional typicality. First, objects and situations should be linked such that objects serve as cues for situations with which they are strongly associated; that is, objects should cue situations in which they are functionally typical and *vice versa* (Barsalou, 2003; Yeh and Barsalou, 2006). We refer to this category of contextual constrain as associations between concepts and contexts (Dieciuc and Folstein, 2019). However, SitCog goes somewhat beyond this first prediction as well. Concepts do not merely serve as cues for generic situations, but should also be integrated into the situation such that causal and spatial relationships between the concept (e.g., of an object like a chicken) and the rest of the situation are somehow represented. Thus, the functional typicality of the object in a particular context is related not only to the strength of the association between object and context, but also to its ability to function in a typical way within the context. For instance, Roth and Shoben (1983) found that milk was more typical than tea in the context of a doughnut shop. One possible reason for this is simply that milk is more strongly associated with a doughnut shop than tea; but another possible reason is that milk tastes better with doughnuts than tea. We refer to this second type of contextual constraint as “pragmatic constraints.” Representation of pragmatic constraints could benefit from experience in a particular domain (e.g., knowing how milk tastes with doughnuts) but also benefits from general knowledge of the world. For instance, Figure 3 shows a toy poodle in a highly atypical context (the surface of the moon). The context is atypical not only because toy poodles are not directly experienced or observed on the moon by anyone, but also because animals cannot breathe in outer space and the poodle would be very cold without a space suit, two facts that most people know from general knowledge. In addition to briefly reviewing evidence supporting pragmatic constraints on typicality, we will also return to the Rumelhart model and evaluate the promise of the model for simulating and accounting for pragmatic constraints.

### Associative Links Between Concepts and Situations

In the previous sections, we discussed evidence supporting two predictions of the CSC framework, arguing specifically that the brain supports both context-independent and context-dependent neural theaters of representational similarity. Now we turn to the predictions of SitCog, outlined above: (1) Objects should serve as cues for scenes in which they are typically observed and *vice versa* and (2) objects should be situated within the scene such that relationships between occupants of the scene are represented and contribute to typicality judgments. The current section evaluates the first of these predictions, for which there is considerable evidence.

As reviewed by Dieciuc and Folstein (2019), the prediction for links between objects and situations is supported by many behavioral findings. Objects are recognized more easily when superimposed on appropriate visual backgrounds (e.g., a football player on a football field) than inappropriate visual backgrounds. (e.g., Palmer, 1975; Davenport and Potter, 2004; Davenport, 2007; Barenholtz, 2013). Contextual features like background scenes (Freeman et al., 2013b) or clothing (Freeman et al., 2011) can exert influence on categorization even when they are task irrelevant. The typicality of perceptual stimulus features and objects has been shown to be dependent on the context in which they are encountered (Roth and Shoben, 1983; Freeman, 2014), as well as the perspective taken by the observer, which can be thought of as an imagined context (Barsalou and Sewell, 1984).

A robust cognitive neuroscience literature, both new and old, demonstrates the neural mechanisms of associative links between concepts and situations. One line of evidence comes from the study of event-related potentials (ERPs), a measure of stimulus-related electrical activity recorded from the scalp. The well-known N400 component of the event-related potential, a centro-parietally distributed negative-going wave peaking about 400 milliseconds after presentation of a word, demonstrates neural sensitivity to sentence context during conceptual access. The N400 is enhanced when, based on the semantic context set up by a sentence, a sentence-final word has an unexpected meaning, but not an unexpected grammatical morpheme or an unexpected font. Decades of research have shown that the N400 is sensitive to the difficulty of semantically integrating a word into a sentence context under normal reading conditions where no special semantic judgment is required (see Kutas and Federmeier, 2011 for a review).

Other work has demonstrated that pictures of objects trigger rapid retrieval of visual contexts in which they are frequently encountered (reviewed in Aminoff et al., 2013). When fMRI activation elicited by pictures of objects with strong contextual associations is compared with weak association objects, a network of areas including the parahippocampal gyrus and retrosplenial cortex is activated, both of which are implicated in scene representation (Bar et al., 2008). Importantly, work with magnetoencephalography (the magnetic cousin of event-related potentials) has shown that these areas become synchronized with other visual areas in the range of 200 milliseconds after stimulus onset, suggesting that contextual information is retrieved quite rapidly and automatically (Kveraga et al., 2011).

Information about the location in which an object is typically found is also present in anterior temporal areas related to semantics. Peelen and Caramazza (2012) instructed participants to make semantic judgments about objects with orthogonal similarity patterns for shape, associated action, and associated location. Multivoxel pattern information about action and location, but not shape, was found in the anterior temporal lobes in locations similar to those observed in studies showing sensitivity to semantic feature similarity (Bruffaerts et al., 2013; Fairhall and Caramazza, 2013; Clarke and Tyler, 2014; Liuzzi et al., 2015; Borghesani et al., 2016; Chen et al., 2016). Finally, medial prefrontal cortex and retrosplenial cortex, two areas implicated in Bar’s work as sensitive to object-scene associations, are also sensitive to the degree of match between an object and background scene. Freeman et al. (2013a) showed that activation in these areas was positively correlated with the “Whiteness” of a morphed face against a Western background and with the “Asianness” of a morphed face against an Asian background^3^.

The literature supporting links between concepts and situations confirms a key prediction of SitCog: objects serve as cues for the retrieval of situations with which they are associated.

*Prima facia*, it might appear that the CSC framework is silent about this prediction or even somewhat counter to it. Whereas SitCog sees the central goal of semantic cognition as selecting which concepts “fit into” particular goals and situations, CSC is more concerned with associations between and access to the features of individual concepts. This can be seen clearly in the way the two theories propose to account for typicality. The SitCog framework proposes that the best concept for a particular situation is first selected and represented in working memory along with the situation that it fits into (Barsalou, 2003). The concept at hand (e.g., the stimulus in an experiment or objects that are actually available to the observer) is then compared to the simulated concept to determine typicality (Barsalou, 1987). In contrast, CSC discusses typicality in terms of distance to the central tendency of the entire category considered together (Rogers et al., 2015).

Computational models supporting the CSC framework also do not seem to represent situations or make explicit predictions about links between concepts and situations, focusing again on the features of individual concepts. The Rumelhart model, for instance, receives localist perceptual input and feeds forward to activate associated semantic features and recurrent versions of the hub and spoke model (Rogers and Patterson, 2007; Chen et al., 2017) receive input from various modality-specific spokes and produce perceptually grounded semantic features in other spokes *via* the central hub. An informative illustration of this mind-set comes from the Chen et al. (2016) study of similarity representation in ATL, in which “Is tropical” was included as a semantic feature. SitCog would view knowledge that an animal is tropical as indicative of situational information retrieved along with the concept (e.g., a bird sitting in a jungle), but Chen et al. coded it as an encyclopedic feature, implying that the information was fundamentally verbal or factual. In contrast, at least one computational model, supporting the SitCog framework, explicitly represents context to facilitate activation of semantic concepts that are associated with the context (van Dantzig et al., 2011).

Despite the relative absence of situational context from the CSC model, it is our view that the model and the broader framework could account for the data reviewed in this section even with minor modifications. One way to think about the various PDP implementations of the hub and spoke model is in terms of a fundamental commitment to semantic cognition as being based on principles of associationism (Rumelhart and McClelland, 1986). To the degree that the findings reviewed in this section indicate that typicality is calculated based on associative links between concepts and situations, accounting for the results within the same computational framework should not be difficult.

The most obvious modification, hardly a modification at all, would be to make the context in which an object is encountered one of the semantic features in the output, perhaps along with a new context node “LOCATED IN.” Recall the procedure sometimes used in the Rumelhart model called “back propagation to representation,” in which it is possible to activate an output node and find the distributed pattern in the context-insensitive representation layer that most strongly activates it. Once the pattern is found, it is possible to test other semantic properties associated with it, demonstrating for instance that things that “CAN breathe” often “HAVE blood.” Using this same method, one could find the representation for ISa poodle and then feed activation forward to find where poodles are usually located (e.g., New York apartments). This mechanism would predict many of the phenomena reported by Bar and his colleagues and is perhaps incorporated even more naturally in recurrent versions of the network that do not include the control network (Chen and Rogers, 2015; Chen et al., 2017).

In the context of the Rumelhart model, other modifications could be possible, for instance modifying relational context nodes of the Rumelhart model to be context-specific (e.g., ISa_doughnut_shop_, ISa_midmorning_break_, etc.), or replacing the relational context nodes, which specify the KIND of feature desired from a concept, with context nodes that specify a situation. For instance, instead of ISa, HAS, and CAN, the context nodes could include DONOUGHT SHOP, FOREST, GARAGE, etc. and activate appropriate instantiations of concepts. For instance, if given “hound” as input (in this case interpreted as verbal input) and “FOREST” as a context, “can run” might be activated strongly in the semantic output layer if this property of hounds were frequently observed in this context. The same input in the “FRONT PORCH” context might activate “likes to sleep” more strongly as a contextually appropriate semantic feature. The more general category “bird” given as verbal input might activate “chicken” and other associated features of chickens in the “FARM” context and “owl” in the forest context, reflecting the typicality of chickens and owls for farms and forests, respectively. Whether these modifications are plausible and can expand the range of phenomena that the model can account for remains a topic for future research.

In summary, links between concepts and situations receive little attention from the CSC framework, but are not fundamentally at odds with the theoretical mechanisms posited by CSC. Overall, we expect that modulation of typicality by perspective, social context, and situational context is accomplished by the control network, suggesting that these effects should be reduced in patients with SA. At the same time, these findings pose a rich set of unanswered questions for the CSC framework, including how situation-appropriate concepts are selected and which frontoparietal control areas are responsible.

### Evidence for Neural Representations of Pragmatic Constraints on Typicality

In the previous section, we argued that, as predicted by SitCog, concepts serve as cues for strongly associated situations in which they are typically found and that, while this is not a strong prediction of the CSC framework, it is not difficult for the CSC framework to account for these findings. We now turn to a second prediction of SitCog that is potentially more problematic for CSC: representation of concepts embedded in situations. By this, we mean not just co-activation of a concept with a scene or situation, but representation of the concept within the situation, including causal relationships with other concepts, including the observer (e.g., Murphy and Medin, 1985). This embeddedness and interrelatedness is one critical aspect of what is referred to in SitCog as a “situated simulation^4^”. Situated simulations provide an account of earlier work suggesting that typicality was driven not only by feature sharing, but the usefulness of a concept for achieving a particular goal, also called the “idealness” of the concept (Barsalou, 1985).

Medin and colleagues showed in several studies that expert populations (such as landscapers and biologists, two kinds of tree experts) and non-Western cultures often use ideals rather than central tendency to determine typicality ratings and that different cultures have different ideals for the same categories (Lynch et al., 2000; Burnett et al., 2005; see Dieciuc and Folstein, 2019 for a more detailed discussion). According to this research, ideals influence typicality by comparing exemplars to the best possible member of that category that would be most effective at fulfilling that category’s functional purpose (e.g., the *best* possible student imaginable) rather than by comparing exemplars to the central tendency of the category (e.g., the *average* student). Ideals also determine typicality in newly constructed (“*ad hoc*”) categories, such as “ways to escape getting assassinated by the mafia” and “places to shop for an antique couch.” *Ad hoc* categories are hypothesized to be created “on the fly” as needed for emergent situations and might not always be represented in long-term memory at all (Barsalou, 1983, 1987).

Creation of these “temporary” categories seems to require mechanisms that go beyond feature sharing or mere strength of association. To figure out how to avoid assassination by the mafia, for instance, one must imagine how a Mafioso might try to find their target and what the target must do as a countermeasure. A “typical” way to avoid assassination would presumably be the one that seemed most effective or most easily came to mind. This involves simulation of causal interactions that are very different from retrieving what kind of bird is most often found on a farm (i.e., a chicken).

In looking for cognitive neuroscience evidence for this aspect of SitCog, we wanted to find papers that went beyond reactivation of modality-specific features (e.g., Simmons et al., 2005; Martin, 2016) because this is an area where SitCog and CSC agree. Instead we wanted to find cognitive neuroscience studies that provided evidence for representation of spatial and causal relationships between the retrieved concept and specifically retrieved visuospatial contexts that might qualify as “situation models” (Zwaan, 2016). Further, we hoped to find evidence for different degrees of “fit” of concepts within the situation models that might be indicative of differences in typicality, similar to Bar’s findings that objects activate the PPA only if they are strongly associated with particular contexts. Perhaps not surprisingly, we could find no experiments that met this high bar and we find it likely that evidence relating to typicality in *ad hoc* and other ideal-based categories is mostly behavioral. One possible reason for this could be that fMRI methods like representational similarity analysis and support vector machines require similarity relationships between stimuli that are relatively stable across participants. Even if situated simulations produce typicality judgments about particular target objects that are stable across participants (Yeh and Barsalou, 2006), the simulations employed by particular participants in order to arrive at typicality judgments could differ in many ways, complicating predictions for RSA. In contrast, RSA is perfect for evaluating overlap between stable semantic features, explaining the abundance of recent evidence for structural typicality reviewed above.

Interestingly, the CSC framework does offer one piece of evidence regarding *ad hoc* categories. Consider the *ad hoc* category “things that could be used to kill a fly (other than a fly swatter).” Corbett et al. (2011) found that, compared to controls, SA patients were particularly impaired when asked to match a concept, like newspaper with a noncanonical use, like killing a fly, suggesting that the control network is important for constructing *ad hoc* categories.

Unfortunately, no model-based simulations of this particular impairment are available, leaving the mechanism by which the control network achieves this an open question.

### The Controlled Semantic Cognition Framework and Goal-Derived Categories

Although little cognitive neuroscience evidence exists for mechanisms of typicality based on ideals and goal-derived categories, these phenomena are supported by a reasonable number of behavioral findings (Barsalou, 1983, 1985; Medin et al., 1997; Lynch et al., 2000; Burnett et al., 2005; but see Kim and Murphy, 2011). We therefore asses here the ability of CSC to account for these phenomena. Ideals and *ad hoc* categories are potentially problematic for the hub and spoke model because resistance to brain damage shown by typical members is explained in terms of proximity to a category’s central tendency, which comes to be represented in the hub over the course of learning. Concepts that are close to the central tendency that share more features with other members are more resistant to “representational distortion” caused by brain damage (Rogers et al., 2015). If ideals were also resistant to anterior temporal lobe damage in SD, it would suggest that something other than shared features represented in the hub might cause robust internal representations.

Ideals are also less easily explained by the computational mechanisms thought to underlie the hub. Whereas the Rumelhart model can naturally predict typicality effects based on central tendency (information about typical exemplars is more easily accessed because it is shared with many similar exemplars), accounting for the use of ideals is less natural, seeming to require greater involvement from a homunculus operating outside the model. One way ideals might be calculated within the Rumelhart model would be to activate candidate exemplars and see if the resulting output features fit some set of goals. Ideal-based typicality of a given exemplar could then be determined *via* activation level of desirable semantic features (e.g., “very strong” or “very fast” for ideal athletes). Selection of candidate exemplars, task goals, and which features are desirable to complete the task would all fall to the homunculus.

Rogers and McClelland (2004) offered explanations for the cross-cultural findings of Medin and colleagues that would preserve central tendency-based accounts of typicality, arguing that they might be caused by a combination of learning and selective-attention-like mechanisms. Landscapers, for instance, encounter the same types of trees repeatedly in similar situations over and over again. To do their job, landscapers must attend to certain attributes, such as “how a tree looks in its surroundings” while biologists attend to “certain distinguishing biological properties” (Rogers and McClelland, 2004, p. 225). This was predicted to result in internal representations with different central tendencies (and therefore different typical exemplars) for different expert groups. Simulations with the model were consistent with this prediction: training on the same categories and features resulted in different internal representations with different central tendencies when different relational contexts were frequently activated during training. Whether explanations like this one truly account for some or all cross-cultural differences remains to be seen, but typicality effects in *ad hoc* categories cannot be explained in this way because *ad hoc* categories are created on the fly rather than through gradual learning.

Overall, whereas there is some evidence that the Rumelhart model can account for typicality in some goal-derived categories – in particular, gradually learned categories associated with culture and expertise – *ad hoc* categories appear to fall outside the scope of computational models in the CSC framework. Other models that learn to represent causal interactions between objects and other visuospatial aspects of situations (e.g., Caligiore et al., 2010) hold promise in accounting for typicality in *ad hoc* categories.

## Conclusion

Focusing on evidence from cognitive neuroscience, this review contrasted two frameworks – the controlled semantic cognition framework and the situated cognition framework – in their ability to account for a range of typicality phenomena that have been observed over several decades. Two types of typicality were considered: (1) typicality related to stable central tendencies of categories, a property of concepts that we refer to as “structural typicality” and (2) typicality that is highly sensitive to context, a property of concepts that we call “functional typicality.”

The CSC framework made two major predictions that were relevant to structural and functional typicality: (1) a context-insensitive neural theater representing stable similarity relationships between concepts and, by implication, stable central tendencies and (2) a context-sensitive neural theater in which similarity relations change depending on what semantic information is required by a task. Regarding the first prediction, there is considerable evidence for a multimodal convergence zone in the ATL that extracts correlations between semantic features and allows access to multimodal concepts. It is quite likely that the ATL represents information about central tendency across category members that has been distilled across many situations. This evidence comes from two major sources. First, studies of SD and SA suggest that damage to the ATL impairs concepts across a wide range of tasks and is sensitive to typicality without need to specifically manipulate context. Second, several recent fMRI studies use the representational similarity analysis technique to demonstrate matches between neural representation in the ATL and behavioral measures of semantic similarity, again without resorting to context manipulation when measuring semantic similarity behaviorally.

Importantly, the degree to which the ATL forms a purely acontextual neural analog to the representation layer in the Rumelhart model or other recurrent models of the “hub” is far less clear. Most studies that have specifically addressed task context have not looked at the ATL as a region of interest and studies of semantic dementia in which the ATL is damaged have rarely manipulated context in a way that addresses particular hypotheses related to the situated cognition framework. Studies that seek to elucidate modulation of information in the ATL by situational and task context would be a welcome addition to the recent wave of studies on the topic and would represent an important bridge between ideas emphasized in CSC and ideas emphasized in SitCog.

Evidence for a context-sensitive neural theater was also reviewed. The CSC framework predicts that selecting information is accomplished by a control network in frontal and parietal areas whose computational analog is thought to be the hidden layer of the Rumelhart model. Representational similarity, and therefore typicality, within the hidden layer is driven by relational context (ISa, HAS, etc.), which can be construed as resulting from the information demands of a task. Recent fMRI data confirm that, like the hidden layer of the Rumelhart model, representational similarity in frontal and parietal areas (and also perceptual areas to some degree) is also driven by task context.

We also evaluated two predictions of SitCog: (1) neural representations of objects should serve as cues for neural representations of strongly associated situations; and (2) retrieved representations of concepts should not only activate modality specific information, but should be “situated” in the sense that spatial and causal relationships between the object and other parts of the situation should be represented. *Ad hoc* categories especially seem to require simulations in which multiple agents interact, with typicality determined by the ease with which a concept functions within this situation.

There was abundant evidence for the first prediction. The parahippocampus, an area associated with representing places and scenes is strongly and rapidly activated by processing of objects with strong contextual associations. The ATL also contains information about where objects are typically located. Overall, these data corroborate the larger prediction that retrieval of associated contexts is a critical aspect of semantic cognition. Regarding the second prediction that semantic cognition should not only involve the retrieval of modality-specific semantic information, but that the information should be situated, we found little evidence in the neural domain and argued that this issue could run up against analysis techniques currently available in fMRI. Thus, the neural correlates of ideals and especially *ad hoc* categories is a potentially rich area for new research.

We also evaluated the ability of the Rumelhart model to account for these two predictions computationally even though no attempt to do so has been made to date. We concluded that the architecture of the Rumelhart model was well suited to represent retrieval of associated contexts but that ideals and *ad hoc* categories appear to pose the strongest challenge to the CSC framework. The main challenge posed by ideals and *ad hoc* categories is that they cannot be represented in the hub, at least not by the mechanisms proposed by CSC. Ideals are problematic because they do not correspond to central tendency, which is how typicality is represented in the hub. *Ad hoc* categories are problematic because they are created on the fly rather than by gradual associative learning, which is how representations in the hub are learned. Therefore, even if typicality in *ad hoc* categories were derived by similarity to central tendency, it could not be calculated in the hub. Furthermore, the hidden layer of the Rumelhart network, which is sensitive to relational context, offers no obvious solution to the problem of ideals and *ad hoc* categories.

How problematic these issues are for the CSC framework depends partly on what one believes CSC is obligated to explain. On one hand, CSC offers no detailed computational model of *ad hoc* categories and ideals but, on the other hand, we know of no mechanistic model that does. Furthermore, we outlined a possible route by which the Rumelhart model could provide the kernel for a larger model that implements other control mechanisms not yet specified. We also reviewed initial evidence that damage to the control network in SA does impair the ability to create *ad hoc* categories (Corbett et al., 2011). Moving forward, therefore, *ad hoc* categories and possibly ideals offer an important avenue for future research into the nature of the control network. Regarding the particular question of typicality, an important question is whether typicality in *ad hoc* categories is calculated based on representations in the ATL, modality-specific areas, or frontoparietal areas, but the available data strongly suggest the latter.

Overall, our review offers a strong potential for reconciliation between the two views of typicality laid out in the beginning: stable typicality on one hand, based on shared features between concepts, and highly malleable, situation-dependent typicality on the other. In a nutshell, the former, “structural typicality,” corresponds to representations in the ATL, while the latter, “functional typicality,” is calculated within the control network in response to current situations and task demands (Dieciuc and Folstein, 2019). The CSC emerges as a strong overall theory of semantic cognition, but SitCog highlights critical areas for further development of the CSC framework, particularly how information is selected and represented in response to many different kinds of situations. Important questions also remain about the sensitivity of the ATL to context and whether it is modulated by control areas in ways similar to visual cortex, or whether it is a fundamentally static, context-insensitive semantic store.

## Author Contributions

JF and MD developed the ideas in this article together. JF reviewed and interpreted the majority of the cognitive neuroscience and computational modelling literature. A key concept, “structural and functional typicality” was contributed by MD.

### Conflict of Interest Statement

The authors declare that the research was conducted in the absence of any commercial or financial relationships that could be construed as a potential conflict of interest.

## Footnotes

^1^Stimuli are represented many times in the brain in many different ways – for instance in the progression from V1 to V4 to the lateral occipital complex, and on to the hippocampus. We think of neural theater as any of these many discrete stages of representation. We use the term theater as an alternative to “module,” which would imply limits on the degree to which the areas interact.

^2^Peelen and Caramazza (2012), reviewed below, did not find evidence that task context (judge location vs. judge action) modulated ATL representations. However, unlike many studies reviewed below, they did not find task effects in any other brain areas either, suggesting that the particular design used in this study might have lacked sensitivity to this contrast.

^3^In Freeman’s study, medial prefrontal cortex was labeled as orbitofrontal cortex, but the coordinates and pairing with retrosplenial cortex suggest a very similar area to that observed by Kveraga et al. (2011).

^4^Modality specificity is another critical aspect of situated simulations that has been strongly emphasized in recent years (Barsalou, 2016). We do not emphasize it here because modality specificity of semantic representation has already been internalized by many theories of semantic knowledge, including CSC (Martin, 2016; Lamdon Ralph et al., 2017; but see Leshinskaya and Caramazza, 2016).
